# Prevalence and Outcomes of Frailty in Korean Elderly Population: Comparisons of a Multidimensional Frailty Index with Two Phenotype Models

**DOI:** 10.1371/journal.pone.0087958

**Published:** 2014-02-04

**Authors:** Hee-Won Jung, Sun-Wook Kim, Soyeon Ahn, Jae-Young Lim, Ji-Won Han, Tae-Hui Kim, Ki-Woong Kim, Kwang-il Kim, Cheol-Ho Kim

**Affiliations:** 1 Department of Internal Medicine, Seoul National University Bundang Hospital, Seongnam, Republic of Korea; 2 Medical Research Collaborating Center, Seoul National University Bundang Hospital, Seongnam, Republic of Korea; 3 Department of Rehabilitation, Seoul National University Bundang Hospital, Seongnam, Republic of Korea; 4 Department of Neuropsychiatry, Seoul National University Bundang Hospital, Seongnam, Republic of Korea; 5 Seoul National University College of Medicine, Seoul, Republic of Korea; 6 Department of Brain and Cognitive Science, Seoul National University College of Natural Sciences, Seoul, Republic of Korea; Medical University of Graz, Austria

## Abstract

**Background:**

Frailty is related to adverse outcomes in the elderly. However, current status and clinical significance of frailty have not been evaluated for the Korean elderly population. We aimed to investigate the usefulness of established frailty criteria for community-dwelling Korean elderly. We also tried to develop and validate a new frailty index based on a multidimensional model.

**Methods:**

We studied 693 participants of the Korean Longitudinal Study on Health and Aging (KLoSHA). We developed a new frailty index (KLoSHA Frailty Index, KFI) and compared predictability of it with the established frailty indexes from the Cardiovascular Health Study (CHS) and Study of Osteoporotic Fracture (SOF). Mortality, hospitalization, and functional decline were evaluated.

**Results:**

The prevalence of frailty was 9.2% (SOF index), 13.2% (CHS index), and 15.6% (KFI). The criteria from CHS and KFI correlated with each other, but SOF did not correlate with KFI. During the follow-up period (5.6±0.9 years), 97 participants (14.0%) died. Frailty defined by KFI predicted mortality better than CHS index (c-index: 0.713 and 0.596, respectively; *p*<0.001, better for KFI). In contrast, frailty by SOF index was not related to mortality. The KFI showed better predictability for following functional decline than CHS index (area under the receiver-operating characteristic curve was 0.937 for KFI and 0.704 for CHS index, p = 0.001). However, the SOF index could not predict subsequent functional decline. Frailty by the KFI (OR = 2.13, 95% CI 1.04–4.35) and CHS index (OR = 2.24, 95% CI 1.05–4.76) were associated with hospitalization. In contrast, frailty by the SOF index was not correlated with hospitalization (OR = 1.43, 95% CI 0.68–3.01).

**Conclusion:**

Prevalence of frailty was higher in Korea compared to previous studies in other countries. A novel frailty index (KFI), which includes domains of comprehensive geriatric assessment, is a valid criterion for the evaluation and prediction of frailty in the Korean elderly population.

## Introduction

Frailty is a state of decreased homeostatic capacity against stress in older adults and reflects physiological age rather than chronological age. By this nature, frailty widely overlaps with functional impairment and comorbidity, but has independent features and prevalence [1]. Moreover, it is an independent risk factor for subsequent mortality, institutionalization, and morbidities such as fall, incontinence, and immobility [2], [3].

Korea is known for being one of the most rapidly aging countries in the world [4]. Therefore, frailty has been a critically important public health problem due to its self-aggravating character and socio-economic burden. However, there is limited data regarding the prevalence and outcome of frailty in Korea. In addition, to our knowledge, there was no validated criterion of frailty for use with the Korean population, although the ‘Korean frailty index’ [5] showed some correlation with Cardiovascular Health Study (CHS) frailty index. Nevertheless, it did not show validity through outcome measures.

Although diverse conceptual models of frailty have been reported, the definition of frailty remains controversial [6]. Previous studies presented various diagnostic criteria for frailty [1], [2], [7], [8], ranging from a simple question appropriate for busy clinical settings to complex models more suitable for research. There have been attempts to diagnose frailty through phenotype model [1]. On the other hand, a model of the accumulation of deficits [8] has been advocated by another group. Recently, a multidimensional model of frailty (Multidimensional Prognostic Index, MPI) showed substantial validity [9].

The phenotype model of CHS frailty index was originally constructed from an epidemiological study and contains fortuitously chosen items from CHS study such as an activity questionnaire that is not typically used in clinical geriatric evaluation. Two later models for frailty have a quantitative feature and use information acquired from a comprehensive geriatric assessment (CGA). The accumulation of deficits model emphasizes the connected features of clinical deficits [10], assuming that stability is established by increasing the number of variables. This model is known for its strong predictability for mortality and functional outcome in a community setting.

On the other hand, MPI was composed of variables from CGA, which has significant predictability for mortality [9]. The MPI was superior to the accumulation of deficit model and operational criteria in predicting the mortality of hospitalized patients [9]. Nevertheless, the validity of the MPI was less clear for functional outcome.

On these backgrounds, we comprehensively evaluated the prevalence and adverse outcomes of frailty in community-dwelling older adults in Korea using established frailty criteria. Furthermore, we developed a novel frailty index which has the characteristics of MPI with variables from CGA, and compared the predictability for clinical outcome from this novel index with the existing phenotypic frailty criteria.

## Materials and Methods

### Study Subjects

The Korean Longitudinal Study on Health and Aging (KLoSHA) has been described in detail elsewhere [11], [12]. Briefly, KLoSHA was a population based prospective cohort study of 992 Koreans 65 years or older residing in the city of Seongnam. The KLoSHA subject group consists of 714 randomly sampled individuals 65 years or older reflecting general elderly population and 278 volunteers 85 years or older. The baseline study was conducted from September 2005 to September 2006 in the Seoul National University Bundang Hospital (SNUBH). This study was conducted according to the Declaration of Helsinki. The Institutional Review Board of SNUBH approved the study protocol. Written informed consent was obtained from all subjects.

Among the 992 subjects, 693 participants underwent evaluation including the Short Physical Performance Battery (SPPB) and the handgrip strength test. Of the 693 subjects, 442 participants completed the 5-year follow up evaluation from May 2010 through March 2012. The reasons for follow up loss were unavailable contact (N = 17), refusal of recommended follow-up tests (N = 144), and death before follow up evaluation (N = 90).

The baseline characteristics of the subjects who lost to follow up were compared with the subjects who completed the follow up. Subjects who lost to follow up were older, more frail, cognitively worse, and more functionally dependent.

### Baseline Geriatric Examination

The baseline examination of KLoSHA includes a broad range of geriatric evaluations. The complete list of evaluation items has been described previously [12].

#### Functional status

Activities of daily living (ADL) were assessed by the Korean Activities of Daily Living (K-ADL) scale, which includes evaluation of dressing, bathing, eating, getting out of bed, and using the toilet, with score ranging from 7 to 21 points. The instrumental ADL was evaluated using the Korean Instrumental Activities of Daily Living (K-IADL) scale, from 0 to 10 points, concerning grooming, housework, meal preparation, bathing, going to places outside the home, using transportation, shopping, managing money, and making phone calls.

#### Body composition, physical activity and performance

To assess body composition, a bioimpedence analysis with Inbody 3.0 (Biospace, Seoul, Korea) was performed with measurements of height and weight. Physical activity was measured by the Baltimore Longitudinal Study of Aging (BLSA) Activities Questionnaire [13]. Physical function was measured by the SPPB [14]. Isometric grip strength was assessed using a handgrip dynamometer (JAMAR hydraulic hand dynamometer, Sammons Preston, Bolingbrook, IL, USA).

#### Cognition and mood

Among many scales used in KLoSHA, the authors used the Korean version of the Geriatric Depression Scale (GDS-K), the Center for Epidemiological Studies depression (CES-D) scale [15], and the Korean Mini-Mental State Examination (K-MMSE) [16].

### Frailty Indexes

#### CHS frailty index

We used a modified version of the original CHS frailty index (CHS index) [1], which has been well validated. Frailty was defined by the following components: (1) unintentional weight loss of 3 kg or more for 6 months; (2) weakness of handgrip strength, ranking in the lowest quintile of the study population adjusted for body mass index; (3) exhaustion, shown by answers to the CES-D scale questions “I felt that everything I did was an effort” and “I could not get going” that were rated “moderate amount to most of the time during the last week”; (4) slowness, determined by a usual walking speed of 0.6 m/s or less; and (5) low physical activity, ranked in the lowest quintile (400 or less Kcal per week) on the BLSA Activities Questionnaire. The subjects having a score of 0 were considered robust, 1–2 indicated prefrail, and ≥3 indicated frail condition.

#### Study of osteoporotic fracture (SOF) frailty index

A modified SOF frailty index (SOF index) [2] was used with the following components: (1) unintentional weight loss of 3 kg or more for 6 months; (2) inability to stand from a chair 5 times; (3) reduced energy level indicated by answering “no” to the question “Do you feel full of energy?” on the GDS-K. Subjects having none of these components were considered to be robust, those having 1 component were considered to be prefrail, and those having 2–3 components were considered to be frail.

#### KLoSHA frailty index

We intended to develop a multidimensional frailty index to reflect domains of CGA including physical function, physical performance, cognitive function, mood, and nutritional status. To increase dynamic range and discriminability of the new frailty index, applying weighting for each domain by its clinical significance was planned, rather than simple summation used in accumulation of deficits model. Accordingly, we selected the variables in KLoSHA that reflect these domains and show statistical significance for predicting mortality in the preliminary analysis. Among the variables, SPPB, K-MMSE, serum albumin level, K-ADL and K-IADL were selected. On the other hand, GDS-K was excluded because it failed to show statistical significance for mortality by neither continuous nor categorical fashion. The cut-off points for the K-MMSE [10] score were adapted from the literature. The weighting values of each variable were defined using the coefficients of the Cox proportional hazard model. Considering the intrinsic redundancy of the frailty model, unadjusted coefficients were used. A KLoSHA frailty index (KFI), total score of 1, was made (Table 1,2). Thereafter, cut-off values for prefrail (≥0.2) and frail (≥0.35) were defined by distributions of frailty index and clinical frailty scale of another study [8] for comparisons between KFI with other phenotype models of frailty.

**Table 1:** Predictability of individual variables for death.

		β	HR	95% CI
12-total SPPB score		0.22	1.24	1.17–1.32
K-ADL score		0.19	1.20	1.10–1.31
K-IADL score		0.25	1.28	1.21–1.36
Low albumin	0–4 g/dL	0.78	2.18	1.46–3.25
K-MMSE score	21–24	0.22	1.24	0.73–2.10
	18–20	0.14	1.15	0.52–2.58
	11–17	1.35	3.85	2.22–6.68
	1–10	2.39	10.92	5.56–21.45

Abbreviations: HR-hazard ratio, 95% CI–95% confidence interval, SPPB-Short Physical Performance Battery, K-ADL score-Korean Activity of Daily Living score, K-IADL score-Korean Instrumental Activity of Daily Living score, K-MMSE score-Korean version of the Mini-Mental Status Examination score.

**Table 2:** Composition of the KLoSHA frailty index.

	Scoring	Value	Weighting	Score
SPPB	12-total SPPB score	12	0.217	2.604
K-ADL score	Total score	21	0.185	3.885
K-IADL score	Number of impaired components	10	0.248	2.480
K-MMSE score	0 (25–), 0.25 (21–24), 0.5 (18–20), 0.75 (11–17), 1 (–10)	1	2.391	2.391
Albumin	1 (0–4 g/dL), otherwise 0	1	0.780	0.780
Divide				12.140
Total				1.000

Abbreviations: SPPB-Short Physical Performance Battery, K-ADL score-Korean Activity of Daily Living score, K-IADL score-Korean Instrumental Activity of Daily Living score, K-MMSE score-Korean version of the Mini-Mental Status Examination score.

### Outcome Measures

All of the participants were flagged for mortality at the National Statistical Office of Korea, which provided data for the date and cause of all deaths occurring until the end of December 2011. We added the mortality data from National Statistical Office of Korea to our dataset using each individual identifier. Functional decline at follow-up was defined as an increment in the K-ADL score. The data on hospitalization after the initial examination was gathered by patient interview at the follow-up assessment.

### Statistical Analysis

We used an independent t-test for continuous variables and χ2 test for discrete variables to evaluate the characteristics of the participants. Cox proportional hazards models were used to assess the associations between variables and death. The appropriateness of the Cox models was checked by log-log plot. The weighting factor of the KFI was derived from the coefficients of the Cox proportional hazards models. The variables included in fully adjusted Cox proportional hazards models were selected from the baseline data. We used Harrell’s c-index [17] for each frailty index to compare the capability of discrimination for mortality. For KFI, bootstrapping of the total study sample was performed 1000 times, and the c-index was calculated. The linear correlation between each index was assessed with Spearman’s coefficient. The effect of frail status on subsequent hospitalization and functional decline was evaluated by logistic regression analysis. Receiver operating characteristic (ROC) curves were made with each index and compared for functional decline and hospitalization. The statistical analysis was performed using STATA 12.0 (StataCorp, College Station, TX, USA).

## Results

### Baseline Characteristics and Frailty Status by each Index

The baseline characteristics, including the anthropometric information, comorbidity, and functional status of 693 subjects, are shown in table 3. The mean age was 75.9 (SD = 8.9) years, and 352 subjects (50.8%) were female. Among the subjects, 143 (20.6%) were oldest-old (≥85 years old), 117 (16.9%) were old-old (75–84 years old), and 433 (62.5%) were young-old (65–74 years old).

**Table 3:** Comparisons of demographic, anthropometric, and laboratory data in examinees between dead or alive during the follow-up period.

	Alive	N = 596	Dead	N = 97	*p*-value
Age (years)	73.39	(7.66)	82.15	(8.82)	**<0.001**
Sex (Female)	311	(52.20)	41	(42.30)	0.070
Body mass index(Kg/m2)	24.25	(3.13)	23.00	(3.48)	**0.001**
Height (Cm)	157.68	(0.05)	158.36	(9.68)	0.530
Muscle mass (Kg)	40.04	(7.62)	39.49	(8.53)	0.551
ASM/ht^2^ (Kg/m2)	11.97	(1.23)	11.67	(1.45)	0.081
Systolic BP (mmHg)	132.30	(17.16)	133.54	(19.87)	0.563
Diastolic BP (mmHg)	83.22	(10.47)	82.38	(12.48)	0.476
Cerebrovascular disease	58	(9.70)	10	(10.3)	0.859
Cardiovascular disease	276	(46.30)	47	(48.50)	0.695
Cancer history	42	(7.00)	9	(9.30)	0.435
Hypertension	420	(70.50)	70	(72.20)	0.734
Diabetes	147	(24.70)	28	(28.90)	0.377
Hemoglobin (g/dL)	13.94	(1.43)	13.12	(1.39)	**<0.001**
Albumin (g/dL)	4.13	(0.23)	4.04	(0.29)	**0.004**
Cholesterol (mg/dL)	203.36	(38.25)	192.15	(33.55)	**0.007**
HDL-cholesterol(mg/dL)	60.48	(15.00)	58.99	(15.83)	0.371
Folate (ng/mL)	13.81	(17.29)	11.57	(12.84)	0.222
Hemoglobin A1C (g/dL)	6.05	(0.84)	6.03	(0.86)	0.851
Creatinine (mg/dL)	1.10	(0.23)	1.26	(0.53)	**0.005**
ESR (mm/hr)	18.69	(12.68)	23.45	(15.53)	**0.005**
Ferritin (ng/mL)	118.84	(122.78)	121.63	(93.27)	0.832
C-reactive protein(mg/dL)	0.23	(0.72)	0.26	(0.57)	0.728
Bilirubin (mg/dL)	0.84	(0.37)	0.82	(0.40)	0.757
ALT (mg/dL)	23.29	(18.17)	18.58	(10.47)	**0.014**
AST (mg/dL)	26.21	(16.74)	24.59	(7.89)	0.354
ALP (mg/dL)	75.68	(24.06)	84.59	(32.76)	**0.012**
SPPB, Balance	3.44	(0.97)	2.78	(1.34)	**<0.001**
SPPB, Walking speed	2.93	(1.03)	2.29	(1.16)	**<0.001**
SPPB, Seat standing	2.73	(1.20)	2.01	(1.24)	**<0.001**
12-total SPPB score	2.78	(2.58)	4.91	(3.13)	**<0.001**
K-ADL score	7.11	(0.74)	7.60	(1.92)	**0.016**
K-IADL score	12.37	(3.96)	15.77	(5.60)	**<0.001**
GDS-K score	10.77	(7.26)	12.01	(6.69)	**0.125**
K-MMSE score	24.17	(4.10)	20.63	(6.50)	**<0.001**
Education (years)	7.81	(5.68)	7.25	(5.65)	0.371
CHS frailty index	1.19	(1.06)	1.72	(1.01)	**<0.001**
SOF frailty index	0.69	(0.64)	0.59	(0.63)	0.457
KLoSHA frailty index	0.25	(0.10)	0.34	(0.15)	**<0.001**

Abbreviations: ASM/ht^2^-appendicular skeletal muscle mass per square meter height, BP-blood pressure, HDL-cholesterol-high density lipoprotein cholesterol, ESR-erythrocyte sedimentation rate, ALT-alanine aminotransferase, AST-aspartate aminotransferase, ALP-alkaline phosphatase, SPPB-Short Physical Performance Battery, K-ADL score-Korean Activity of Daily Living score, K-IADL score-Korean Instrumental Activity of Daily Living score, GDS-K score-Korean version of the Geriatric Depression Scale score, K-MMSE score- the Korean Mini-Mental State Examination score, CHS-Cardiovascular Health Study, SOF-Study of Osteoporotic Fracture, KLoSHA-Korean Longitudinal Study on Health and Aging.

Data are presented as the mean (SD) or number (%).

Among the 621 subjects who were evaluated for the CHS index, 82 (13.2% total; 19.2% female, 7.3% male) were frail, and 369 (59.4% total; 60.6% female, 58.3% male) were prefrail. In the SOF index of 663 subjects, 61 (9.2% total; 9.9% female, 8.5% male) were frail, and 328 (49.5% total; 49.1% female, 49.8% male) were prefrail. The KFI was calculated for 668 subjects, showing 104 frail (15.6% total, KFI≥0.35; 22.2% female, 9.0% male) and 287 prefrail (43.0% total, 0.20≤KFI<0.35; 44.3% female, 41.6% male).

Spearman’s coefficient between the CHS index and the SOF index was 0.252 (*p*<0.001), that between the CHS index and the KFI was 0.487 (*p*<0.001), and that between the SOF index and KFI was −0.003 (*p = *0.949).

During a mean follow-up of 5.57 years (SD = 0.93), 97 subjects died, 139 experienced hospitalization, and 18 showed functional decline. The subjects with follow-up loss were older (*p*<0.001) and more frail at the baseline evaluation (*p*<0.001 with KFI, *p* = 0.001 with CHS index). They had a higher GDS-K score (*p* = 0.001), more impaired K-ADL (*p* = 0.002) and K-IADL (*p<*0.001), and a worse SPPB score (*p<*0.001).

**Figure 1:**
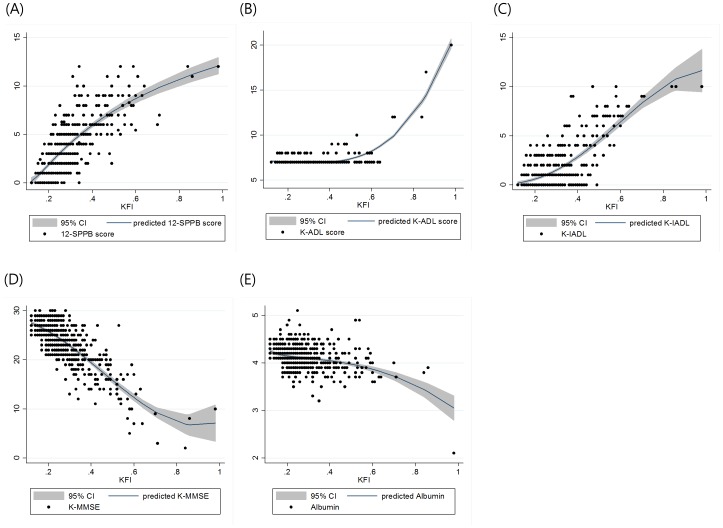
The distributions of each KFI-component variable by KFI and fractional polynomial prediction plots. The shaded area denotes a 95% confidence interval for the prediction curve. (A) 12-SPPB score (B) K-ADL score (C) K-IADL score (D) K-MMSE score (E) albumin (g/dL).

**Figure 2:**
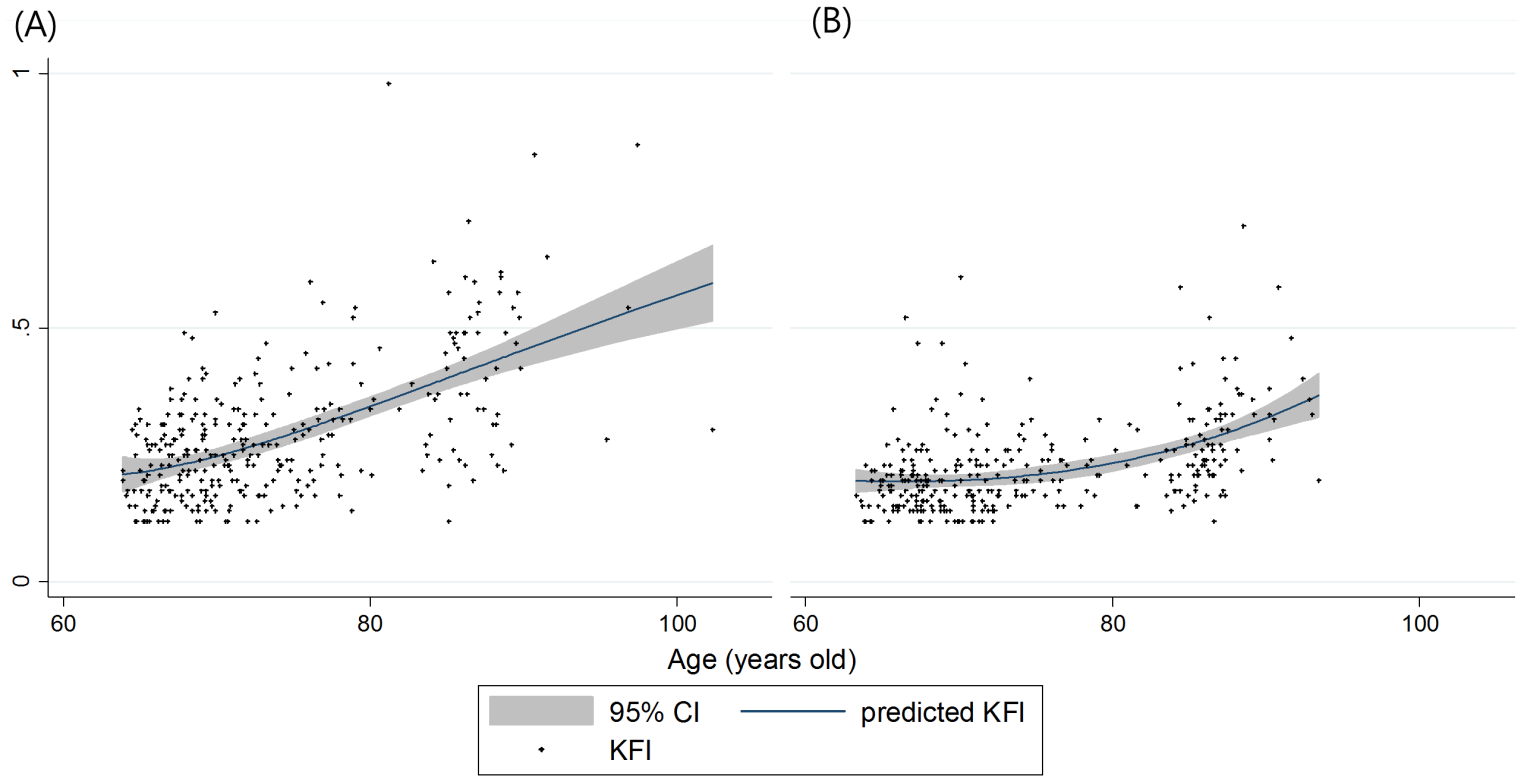
The distributions of KFI by age and fractional polynomial prediction plots plotted by sex, (A) female, (B) male. The shaded area denotes a 95% confidence interval for the prediction curve.

The correlations between KFI and key variables or age are presented in figure 1 and 2, respectively. In the subjects who were classified as frail by the 3 indexes, the SPPB score, K-IADL score, subjective health status, and duration of education were worse (all *p*<0.05). Frail subjects by the CHS index and the KFI were older, had lower muscle mass, albumin levels, and MMSE scores (all *p*<0.05).

### Predictability of Clinical Outcomes by each Index

Outcomes including mortality, functional decline, and hospitalization were evaluated. As shown in table 2, the SPPB score, K-ADL score, low albumin, and K-MMSE were related to mortality. The frailty status defined by the KFI and the CHS index were correlated with mortality, while frailty from the SOF index was not (Table 4). Harrell’s c-indexes of each frailty index are shown in table 5, and there is a statistically significant difference between the KFI and CHS index (*p*<0.001), KFI showing better predictability for mortality.

**Table 4:** Unadjusted and adjusted hazard ratio for the mortality according to the frailty status from each frailty index.

	HR	95% CI
Unadjusted Cox proportional hazard analysis		
KLoSHA index (Frail)	7.23	4.01–13.05
CHS index (Frail)	4.05	1.62–10.16
SOF index (Frail)	0.67	0.28–1.58
Fully adjusted Cox proportional hazard analysis*		
KLoSHA index (Frail)	2.18	1.07–4.45
CHS index (Frail)	1.33	0.50–3.60
SOF index (Frail)	0.69	0.29–1.67

* Adjusted by age, hemoglobin, cholesterol, creatinine, erythrocyte sedimentation rate, alanine aminotransferase, alkaline phosphatase.

Reference variable: Robust state of each frailty index.

Abbreviations: HR-hazard ratio, 95% CI-95% confidence interval, KLoSHA-Korean Longitudinal Study on Health and Aging, CHS-Cardiovascular Health Study, SOF-Study of Osteoporotic Fracture.

**Table 5:** Comparisons of predictability for mortality by frailty status from each frailty index.

	C-index	*p*-value†	95% CI
KLoSHA index*	0.713		0.656–0.770
CHS index	0.596		0.549–0.642
SOF index	0.542		0.485–0.598
KLoSHA index vs. CHS index		<0.001	
KLoSHA index vs. SOF index		<0.001	

* Bootstrapped 1000 times.

†p-value for C-index difference.

Abbreviations: 95% CI-95% confidence interval, KLoSHA-Korean Longitudinal Study on Health and Aging, CHS-Cardiovascular Health Study, SOF-Study of Osteoporotic Fracture.

**Figure 3:**
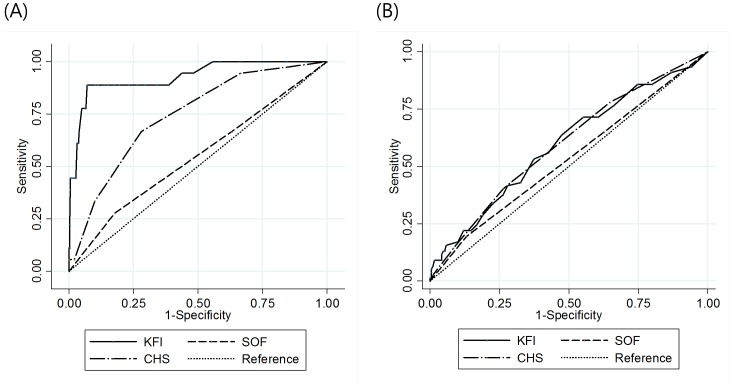
ROC curves for (A) functional decline, (B) hospitalization. (A) The area under the curve is 0.937 for KFI, 0.704 for CHS, and 0.565 for SOF; for the difference of each ROC curve, *p* = 0.001 for KFI and CHS, *p*<0.001 for KFI and SOF, *p* = 0.069 for CHS and SOF. (B) The area under the curve is 0.543 for KFS, 0.560 for CHS, and 0.505 for SOF; for the difference of each ROC curve, *p* = 0.639 for KFI and CHS, *p* = 0.374 for KFI and SOF, *p* = 0.180 for CHS and SOF.

Functional decline was analyzed with the frailty index using a logistic regression model. Frailty assessed by the KFI and CHS indexes predicted subsequent functional decline, which was defined by 1 or more increments on the K-ADL score (Table 6). The association between the frailty index and hospitalizations was analyzed using a logistic regression model. Frailty from the KFI and CHS index was related to a subsequent hospitalization (Table 7). The ROC curves for functional decline and hospitalization by each index are shown in figure 3. The KFI had a significantly larger area under the curve (AUC) for functional decline compared to the CHS and SOF indexes (AUC of ROC curve: 0.937, 0.704 and 0.565, respectively. Comparing each ROC curve, *p* = 0.001 between KFI and CHS, *p*<0.001 between KFI and SOF, *p* = 0.069 between CHS and SOF). The KFI and CHS showed no significant difference in the prediction of subsequent hospitalization.

**Table 6:** Impact of frailty status from each frailty index on subsequent functional decline.

		OR	95% CI
KLoSHA index			
	Prefrail	4.06	0.42–39.39
	Frail	148.00	18.54–1181.72
CHS index			
	Prefrail	6.62	0.85–51.87
	Frail	20.53	2.40–175.80
SOF index			
	Prefrail	0.47	0.17–1.32
	Frail	0.81	0.17–3.82

Reference variable: Robust state of each frailty index.

Abbreviations: OR-odds ratio, 95% CI-95% confidence interval, KLoSHA-Korean Longitudinal Study on Health and Aging, CHS-Cardiovascular Health Study, SOF-Study of Osteoporotic Fracture.

**Table 7:** Impact of frailty status from each frailty index on following experience of hospitalization.

		OR	95% CI
KLoSHA index			
	Prefrail	0.90	0.57–1.41
	Frail	2.13	1.04–4.35
CHS index			
	Prefrail	1.44	0.87–2.39
	Frail	2.24	1.05–4.76
SOF index			
	Prefrail	0.94	0.60–1.47
	Frail	1.43	0.68–3.01

Reference variable: Robust state of each frailty index.

Abbreviations: OR-odds ratio, 95% CI-95% confidence interval, KLoSHA-Korean Longitudinal Study on Health and Aging, CHS-Cardiovascular Health Study, SOF-Study of Osteoporotic Fracture.

## Discussion

In this study, we developed a novel frailty index (KFI) scoring from 0 to 1. To the best of author’s knowledge, applying weighting factors for an existing CGA component to create a frailty index is a novel approach. We also showed the prevalence and clinical outcomes of frailty, using existing and novel criteria among a population of Korean community-dwelling elderly people. The KFI showed better predictability in mortality and functional decline compared with the CHS index.

The prevalence of frailty in this study is higher than that in other studies conducted with Caucasian and Asian [18] populations. In the original CHS study [1], the prevalence of frailty was 7.3% for females and 4.9% for males. A study [19] conducted in China used a frailty index ranging from 0 to 1, and showed a frailty index pattern by age and sex similar to that in this study. Because the KLoSHA consists of 22% of volunteers aged 85 or older, the study population in this study is generally older than that in other cohort studies and may show an increased prevalence of frailty.

An effective frailty index should have the following characteristics [8]. It should be useful, simple, and brief for use in a clinical setting. The domains and characteristics of the KFI were originally chosen to enable automatic calculation from a previously prepared electronic medical record based on CGA data. Although it is cumbersome for primary physicians to use as brief screening test, clinicians can automatically obtain the KFI with CGA and predict the physiological age and quantified vulnerability of a patient.

A good frailty index should show a general demographic pattern of frailty. Using the KFI in KLoSHA, a gradually rising frailty index was observed with increasing age. The KFI of females was greater than the age-matched KFI of males and never crossed (Figure 2). This phenomenon of more prevalent vulnerability in females is observed in many other studies of frailty [19], [20]. Frailty has features independent of functional impairment or comorbidity, although it is broadly correlated with them. The KFI, ADL, and IADL share significant proportions, having vectors of functional impairment that reflect the phenotypic characteristics of frailty.

An effective frailty index may predict adverse outcomes, such as mortality, institutionalization, and functional decline. Using c-statistics, the predictive validity for mortality by the KFI is better than that from the CHS index, which has been validated effectively by larger studies [1], [2], [21]. Interestingly, CHS index lost its predictability for mortality in a fully-adjusted Cox proportional hazards model. By comparing the ROC curves for functional decline and hospitalization of the three indexes, the KFI predicted functional decline better than CHS index, and the KFI prediction for hospitalization was comparable with the prediction of the CHS index.

Although the SOF index was significantly correlated with the CHS index, it could not predict mortality, functional decline, and hospitalization contrary to results from previous studies [2], [9]. In a search by the author, there was no report showing the validity of the SOF index in the Asian population. In this study, significant weight loss of 3 kg for 6 months was not related to mortality using the Cox-proportional hazard analysis (HR = 0.912, 95% CI 0.713–1.167, *p* = 0.465). Furthermore, only 22 subjects were unable to stand up from their seat 5 times without using their arms. Although the CHS index included the weight loss item, it showed superior predictability for outcome compared to the SOF index. This phenomenon may be explained by the low model stability of the SOF index because it is limited to three components. These findings indicate that the variables chosen in the SOF index may not be appropriate for the elderly in Korea. Accordingly, clinicians should be cautious adopting frailty indexes validated in Caucasians for use with other ethnic groups.

Similar to other major studies of frailty, KLoSHA was not originally designed to assess frailty. Therefore, not all of domains routinely measured in CGA were included in KLoSHA. For this reason, authors used SPPB for physical performance and serum albumin level for nutritional status rather than originally intended timed get up and go test and mini-nutritional assessment (MNA), which can be acquired from CGA. Because SPPB is not usually performed as a part of CGA, further studies are needed to replace SPPB with another easier method such as timed get up and go test or usual gait speed for universal application of KFI.

This study has several limitations. There were many follow-up losses, especially for the 5-year follow-up examination, with poorer baseline characteristics identified in these subjects. Although we used complete government registry data for death occurrences, the unbalanced follow-up losses may weaken the validity of the follow-up data for functional status and hospitalization. Furthermore, follow-up examination was limited to 5 year later from baseline evaluation, impeding opportunity to assess geriatric outcomes including fall down, time of functional decline, and institutionalization. Due to the relatively small sample size, bootstrapping was used to cross-validate the c-index for the KFI, rather than a validation cohort. Consequently, the problem of over fitting cannot be completely ruled out. In addition, we used the unadjusted coefficients from Cox proportional hazards model as the weighting value for each component of KFI. Accordingly, this method might compromise the generalizability of the study findings to other population. Another external validation study for KFI is needed to solve this limitation. Nevertheless, KFI predicted functional decline better than CHS index and also could predict hospitalization, showed performance beyond its expected data-driven ability for mortality prediction.

In conclusion, we showed the prevalence and outcome of frailty in Korea. Also, we devised a new multidimensional frailty index, which includes domains from the CGA. By comparisons with previously developed phenotype models, the KFI showed its validity as appropriate frailty assessment instrument for Korean elderly population.

## References

[1] Fried LP, Tangen CM, Walston J, Newman AB, Hirsch C, et al (2001) Frailty in older adults: evidence for a phenotype. J Gerontol A Biol Sci Med Sci 56: M146–156. 11253156 10.1093/gerona/56.3.m146

[2] Ensrud KE, Ewing SK, Taylor BC, Fink HA, Cawthon PM, et al (2008) Comparison of 2 frailty indexes for prediction of falls, disability, fractures, and death in older women. Arch Intern Med 168: 382–389. 18299493 10.1001/archinternmed.2007.113

[3] Sternberg SA, Wershof Schwartz A, Karunananthan S, Bergman H, Mark Clarfield A (2011) The identification of frailty: a systematic literature review. J Am Geriatr Soc 59: 2129–2138. 22091630 10.1111/j.1532-5415.2011.03597.x

[4] OECD (2012) OECD Economic Surveys: Korea 2012, OECD publishing. 17p.

[5] Hwang HS, Kwon IS, Park BJ, Cho BL, Yoon JL, et al (2010) The validity and reliability of Korean Frailty Index. J Korean Geriatr Soc 14: 191–202.

[6] Clegg A, Young J, Iliffe S, Rikkert MO, Rockwood K (2013) Frailty in elderly people. Lancet 381: 752–762. 23395245 10.1016/S0140-6736(12)62167-9 PMC4098658

[7] Morley JE, Malmstrom TK, Miller DK (2012) A simple frailty questionnaire (FRAIL) predicts outcomes in middle aged African Americans. J Nutr Health Aging 16: 601–608. 22836700 10.1007/s12603-012-0084-2 PMC4515112

[8] Rockwood K, Song X, MacKnight C, Bergman H, Hogan DB, et al (2005) A global clinical measure of fitness and frailty in elderly people. CMAJ 173: 489–495. 16129869 10.1503/cmaj.050051 PMC1188185

[9] Pilotto A, Ferrucci L, Franceschi M, D'Ambrosio LP, Scarcelli C, et al (2008) Development and validation of a multidimensional prognostic index for one-year mortality from comprehensive geriatric assessment in hospitalized older patients. Rejuvenation Res 11: 151–161. 18173367 10.1089/rej.2007.0569 PMC2668166

[10] Searle SD, Mitnitski A, Gahbauer EA, Gill TM, Rockwood K (2008) A standard procedure for creating a frailty index. BMC Geriatr 8: 24. 18826625 10.1186/1471-2318-8-24 PMC2573877

[11] Kim KI, Chang HJ, Cho YS, Youn TJ, Chung WY, et al (2008) Current status and characteristics of hypertension control in community resident elderly Korean people: data from a Korean longitudinal study on health and aging (KLoSHA study). Hypertens Res 31: 97–105. 18360024 10.1291/hypres.31.97

[12] Park JH, Lim S, Lim JY, Kim KI, Han MK, et al (2007) An overview of the Korean Longitudinal Study on Health and Aging. Psychiatry Investigation 4: 84–95.

[13] McGandy RB, Barrows CH Jr, Spanias A, Meredith A, Stone JL, et al (1966) Nutrient intakes and energy expenditure in men of different ages. J Gerontol 21: 581–587. 5918313 10.1093/geronj/21.4.581

[14] Guralnik JM, Simonsick EM, Ferrucci L, Glynn RJ, Berkman LF, et al (1994) A short physical performance battery assessing lower extremity function: association with self-reported disability and prediction of mortality and nursing home admission. J Gerontol 49: M85–94. 8126356 10.1093/geronj/49.2.m85

[15] Jung IK, Kwak DI, Joe SH, Lee HS (1997) A Study of Standardization of Korean Form of Geriatric Depression Scale (KGDS). J Korean Geriatr Psychiatry 1: 61–72.

[16] Kang Y, Na DL, Hahn S (1997) A validity study on the Korean Mini-Mental State Examination (K-MMSE) in dementia patients. J Korean Neurol Assoc 15: 300–308.

[17] Harrell FE (2001) Regression modeling strategies : with applications to linear models, logistic regression, and survival analysis. New York: Springer. xxii, 568p.

[18] Imuta H, Yasumura S, Abe H, Fukao A (2001) The prevalence and psychosocial characteristics of the frail elderly in Japan: a community-based study. Aging 13: 443–453. 11845972

[19] Dupre ME, Gu D, Warner DF, Yi Z (2009) Frailty and type of death among older adults in China: prospective cohort study. British Medical Journal 338: b1175. 19359289 10.1136/bmj.b1175 PMC2667569

[20] Kulminski AM, Ukraintseva SV, Akushevich IV, Arbeev KG, Yashin AI (2007) Cumulative index of health deficiencies as a characteristic of long life. J Am Geriatr Soc 55: 935–940. 17537097 10.1111/j.1532-5415.2007.01155.x PMC1893090

[21] Bandeen-Roche K, Xue QL, Ferrucci L, Walston J, Guralnik JM, et al (2006) Phenotype of frailty: characterization in the women's health and aging studies. J Gerontol A Biol Sci Med Sci 61: 262–266. 16567375 10.1093/gerona/61.3.262

